# The Neomycin Resistance Cassette in the Targeted Allele of *Shank3B* Knock-Out Mice Has Potential Off-Target Effects to Produce an Unusual Shank3 Isoform

**DOI:** 10.3389/fnmol.2020.614435

**Published:** 2021-01-11

**Authors:** Chunmei Jin, Hyojin Kang, Taesun Yoo, Jae Ryun Ryu, Ye-Eun Yoo, Ruiying Ma, Yinhua Zhang, Hyae Rim Kang, Yoonhee Kim, Hyunyoung Seong, Geul Bang, Sangwoo Park, Seok-Kyu Kwon, Woong Sun, Hyunkyung Kim, Jin Young Kim, Eunjoon Kim, Kihoon Han

**Affiliations:** ^1^Department of Neuroscience, College of Medicine, Korea University, Seoul, South Korea; ^2^Department of Biomedical Sciences, College of Medicine, Korea University, Seoul, South Korea; ^3^Division of National Supercomputing, Korea Institute of Science and Technology Information, Daejeon, South Korea; ^4^Center for Synaptic Brain Dysfunctions, Institute for Basic Science, Daejeon, South Korea; ^5^Department of Anatomy, College of Medicine, Korea University, Seoul, South Korea; ^6^Department of Biological Sciences, Korea Advanced Institute of Science and Technology, Daejeon, South Korea; ^7^Research Center for Bioconvergence Analysis, Korea Basic Science Institute, Ochang, South Korea; ^8^College of Pharmacy, Korea University, Sejong, South Korea; ^9^Center for Functional Connectomics, Korea Institute of Science and Technology, Brain Science Institute, Seoul, South Korea; ^10^Department of Biochemistry and Molecular Biology, College of Medicine, Korea University, Seoul, South Korea

**Keywords:** *Shank3B* knock-out, Neo cassette, gene expression, off-target effect, mouse chromosome 15

## Abstract

Variants of the SH3 and multiple ankyrin repeat domains 3 (*SHANK3*), which encodes postsynaptic scaffolds, are associated with brain disorders. The targeted alleles in a few *Shank3* knock-out (KO) lines contain a neomycin resistance (Neo) cassette, which may perturb the normal expression of neighboring genes; however, this has not been investigated in detail. We previously reported an unexpected increase in the mRNA expression of *Shank3* exons 1–12 in the brains of *Shank3B* KO mice generated by replacing *Shank3* exons 13–16 with the Neo cassette. In this study, we confirmed that the increased *Shank3* mRNA in *Shank3B* KO brains produced an unusual ∼60 kDa Shank3 isoform (Shank3-N), which did not properly localize to the synaptic compartment. Functionally, Shank3-N overexpression altered the dendritic spine morphology in cultured neurons. Importantly, Shank3-N expression in *Shank3B* KO mice was not a compensatory response to a reduction of full-length Shank3 because expression was still detected in the brain after normalizing the level of full-length Shank3. Moreover, in another *Shank3* KO line (*Shank3* gKO) with a similar *Shank3* exonal deletion as that in *Shank3B* KO mice but without a Neo cassette, the mRNA expression levels of *Shank3* exons 1–12 were lower than those of wild-type mice and Shank3-N was not detected in the brain. In addition, the expression levels of genes neighboring *Shank3* on chromosome 15 were altered in the striatum of *Shank3B* KO but not *Shank3* gKO mice. These results suggest that the Neo cassette has potential off-target effects in *Shank3B* KO mice.

## Introduction

The SH3 and multiple ankyrin repeat domains protein 3 (*SHANK3*) gene encodes core scaffolding proteins that organize the macromolecular protein complex in the postsynaptic density of glutamatergic excitatory synapses (Sheng and Kim, 2000, 2011). Duplications, deletions, and various point mutations of *SHANK3* have been identified in individuals with numerous types of brain disorders, including autism spectrum disorders (ASDs), bipolar disorder, intellectual disability, and schizophrenia, suggesting that Shank3 is critical for proper synaptic development and function (Grabrucker et al., 2011; Guilmatre et al., 2014; Monteiro and Feng, 2017; Ey et al., 2020).

To understand the detailed neurobiological mechanisms underlying *SHANK3*-associated brain disorders, more than 10 different *Shank3* knock-out (KO) mouse lines carrying deletions of different *Shank3* exons (of total 22 exons) have been generated to date, and molecular, cellular, and functional changes in their brains have been characterized (Jiang and Ehlers, 2013; Monteiro and Feng, 2017; Eltokhi et al., 2018). *Shank3* produces multiple Shank3 protein isoforms resulting from intragenic promoters and alternative splicing (Wang et al., 2014; Choi et al., 2015). Therefore, the combination of Shank3 isoforms can differ among *Shank3* KO lines, explaining phenotypic heterogeneity among the KO lines (Monteiro and Feng, 2017).

Moreover, technically, in the targeted alleles of a few *Shank3* KO lines, selection markers for gene targeting in embryonic stem cells, such as the neomycin resistance (Neo) cassette, were not removed (Eltokhi et al., 2018). Importantly, the Neo cassette can affect the expression of neighboring genes in the genome (i.e., off-target effects) (Pham et al., 1996; Scacheri et al., 2001; Meier et al., 2010; West et al., 2016). Therefore, it is possible that the expression levels of the remaining *Shank3* exons as well as neighboring genes on mouse chromosome 15 are affected by the Neo cassette in these *Shank3* KO lines; however, this has not been carefully considered or investigated.

*Shank3B* KO mice (Peca et al., 2011) in which exons 13–16 of the *Shank3* gene are replaced with the Neo cassette are among the most extensively characterized ASD models. We recently identified an unexpected increase in the mRNA expression of *Shank3* exons 1–12 (i.e., 5′ of the Neo cassette) in the brains of *Shank3B* KO mice (Jin et al., 2019a; Lee et al., 2019a). In particular, the mRNA levels of *Shank3* exons 1–12 increased significantly in the cortex, striatum, and hippocampus of both juvenile and adult *Shank3B* KO mice. Furthermore, in multiple brain regions of *Shank3B* KO mice, the increased mRNA of *Shank3* exons 1–12 possibly produced an unusual ∼60 kDa Shank3 protein isoform containing the N-terminal domains of Shank3, which was not expressed in wild-type (WT) brains (Jin et al., 2019a). In a previous study, we speculated that the increase in mRNA levels of *Shank3* exons 1–12 could be due to the compensatory activation of distal *Shank3* promoters in response to reduced Shank3 protein levels in *Shank3B* KO brains (Jin et al., 2019a).

In this study, however, we provide several lines of evidence suggesting that the increased mRNA and protein expression levels of *Shank3* exons 1–12 in *Shank3B* KO brains can likely be attributed to off-target effects of the Neo cassette which is retained in the targeted allele. Moreover, we show that the expression levels of genes neighboring *Shank3* on mouse chromosome 15 differ between *Shank3B* KO brains and WT brains. Together, our results highlight the need for the careful characterization and interpretation of the phenotypes in *Shank3B* KO mice, and potentially other *Shank3* KO lines with the Neo cassette in the targeted allele, especially with respect to gene expression changes.

## Materials and Methods

### Mice

The *Shank3B* KO (Δ13–16), *Shank3* global KO (gKO) (Δ14–16), and enhanced green fluorescent protein (*EGFP*)*-Shank3* transgenic (TG) mice used in this study have been described previously (Peca et al., 2011; Han et al., 2013b; Lee et al., 2017a; Yoo T. et al., 2018; Yoo T. et al., 2019). The mice were bred and maintained on a C57BL/6J (Japan SLC) background according to the Korea University College of Medicine Research Requirements. All procedures were approved by the Committee on Animal Research at Korea University College of Medicine (KOREA-2018-0003). The mice were fed and had access to water *ad libitum* and were housed in group (4–6 mice per cage) under a 12 h light-dark cycle at 18–25°C. All experiments were performed with adult (8–10 weeks old) male mice, unless otherwise specified.

### RNA Purification and Real-Time Quantitative Reverse Transcription PCR (qRT-PCR)

qRT-PCR was performed as described previously (Jin et al., 2018; Zhang et al., 2018; Lee et al., 2019a). Briefly, total RNA was extracted from tissue samples using an miRNeasy Mini Kit (Qiagen, #217004) and 2 μg of total RNA was used for cDNA synthesis using the iScript cDNA Synthesis Kit (Bio-Rad, #BR170-8891). Target mRNAs were detected and quantified by a real-time PCR instrument (CFX96 Touch; Bio-Rad) using SYBR Green Master Mix (Bio-Rad, #BT170-8884AP). The results were analyzed using the comparative Ct method and were normalized against the levels of housekeeping gene *Gapdh* (Han et al., 2013a). The primer sequences for real-time PCR were described previously (Jin et al., 2019a) or are as follows:

Mouse *Gapdh*

Forward 5′-GGCATTGCTCTCAATGACAA-3′
Reverse 5′-CCCTGTTGCTGTAGCCGTAT-3′

Mouse *Acvrl1*

Forward 5′-GGGCCTTTTGATGCTGTCG-3′
Reverse 5′-TGGCAGAATGGTCTCTTGCAG-3′

Mouse *Adamts20*

Forward 5′-GTCCTGGGAAGTTCGTTTCCA-3′
Reverse 5′-GGCTGAAATGCCGGTTCTG-3′

Mouse *Cers5*

Forward 5′-TGCTGTTTGAGCGATTTATTGC-3′
Reverse 5′-GGTTCCACCTTATTGACAGGAC-3′

Mouse *Cpne8*

Forward 5′-ACATTGGGGGAGATTGTTGGT-3′
Reverse 5′-ACTTCTGTCTTGTGGCAAATTGT-3′

Mouse *Dennd6b*

Forward 5′-TTCCGCCCCTACTTCACCAT-3′
Reverse 5′-AGAAAGGGTTTGTGACTCCCA-3′

Mouse *Slc11a2*

Forward 5′-CAATGTCTTTGTCGTGTCCGT-3′
Reverse 5′-GCGACCATTTTAGGTTCAGGAAT-3′

### Biochemical Analysis and Western Blotting

Whole lysates and subcellular fractions of mouse tissue samples were prepared as described previously (Han et al., 2009; Jin et al., 2019a, b). Briefly, for whole lysate preparation, frozen mouse tissues were homogenized in RIPA buffer (50 mM Tris–HCl pH 8.0, 150 mM NaCl, 0.1% SDS, 1% Triton X-100, 0.5% sodium deoxycholate) with freshly added protease and phosphatase inhibitors (Sigma-Aldrich, #04906837001 and #05892970001). The protein concentration was measured using the Bradford Protein Assay (Bio-Rad, #500-0006). The lysate was heated in 1 × NuPAGE LDS sample buffer (Thermo Fisher Scientific, #NP0007) containing 1 × NuPAGE reducing agent (Thermo Fisher Scientific, #NP0004). From each sample, 10–20 mg of protein was loaded onto 4–15% Mini-PROTEAN TGX Precast Protein Gels (Bio-Rad, #4561084) for western blotting. The proteins were transferred to the PVDF Membrane (Millipore, #IPVH00010). Antibodies used for western blot analysis were GAPDH (Cell Signaling, #2118, 1:3,000), Histone H3 (Cell Signaling, #4499, 1:2,000), Homer1b/c (Synaptic Systems, 160-002, 1:1,000), PSD-95 (Thermo Scientific, MA1-046, 1:1,000), and Shank3 N-terminal (aa 192–221, 1:1,000) (Lee J. et al., 2015). Western blot images were acquired using the ChemiDoc Touch Imaging System (Bio-Rad) and quantified using ImageJ software.

### Neuron Culture, Transfection, and Immunocytochemistry

Cultured hippocampal neurons were prepared from embryonic day 18 rat brains as described previously (Lee et al., 2017c, Lee et al., 2019b; Jin et al., 2018). Dissociated neurons on poly-L-lysine–coated coverslips were placed in Neurobasal medium supplemented with B27 (Invitrogen), 0.5 mM L-glutamine, and penicillin/streptomycin (Thermo Fisher Scientific). For immunocytochemistry, cultured hippocampal neurons at days *in vitro* (DIV) 7 were transfected with EGFP alone (pEGFP-N1) or with EGFP and HA-Shank3-N (pCS2 HA-rat Shank3 1–536) constructs using calcium phosphate. The neurons were fixed at DIV 20 with 4% PFA/sucrose, permeabilized with 0.2% Triton X-100, and incubated with GFP (Abcam, ab13970, 1:1,000) and HA (Santa Cruz, sc-7392, 1:500) primary and dye-conjugated secondary antibodies (Jackson ImmunoResearch). Images were acquired by confocal microscopy (Zeiss, LSM800) and quantified using ImageJ software in a blinded manner.

### In-Gel Digestion

Reduction with dithiothreitol and alkylation with iodoacetamide was performed before each gel piece was treated with trypsin to digest the proteins. Gel pieces were washed with distilled water and 100% acetonitrile, swollen in digestion buffer containing 50 mM ammonium bicarbonate and 500 ng trypsin, and then incubated at 37°C for 16 h. Peptides were recovered by two cycles of extraction with 50 mM ammonium bicarbonate and 90% acetonitrile. The resulting peptide extracts from each gel piece were lyophilized and stored at −20°C until mass spectrometry (MS) analysis.

### Liquid Chromatography (LC)–MS/MS Analysis

Peptides were analyzed using a LC–MS/MS system consisting of an Ultimate 3000 HPLC (Thermo Fisher scientific) and an Orbitrap Eclipse mass spectrometer (Thermo Fisher Scientific) equipped with a nano-electrospray source (EASY-Spray Sources; Thermo Fisher Scientific). Peptides were trapped on 75 μm × 2 cm C18 precolumn (nanoViper, Acclaim PepMap100; Thermo Fisher Scientific) before being separated on an analytical C18 column (75 μm × 50 cm PepMap RSLC; Thermo Fisher Scientific) at a flow rate of 250 nl/min. The mobile phases A and B were composed of 0 and 100% acetonitrile containing 0.1% formic acid, respectively. The LC gradient began with 5% B and was stayed at 5% B for 10 min, ramped to 13% B for 50 min, to 25% B for 65 min, to 95% for 5 min, and remained at 95% B over 5 min. Finally, it was ramped to 5% B for another 1 min. The column was re-equilibrated with 5% B for 14 min before the next run. The voltage applied to produce an electrospray was 2,050 V. During the chromatographic separation, the Orbitrap Eclipse was operated in data-dependent mode, automatically switching between MS1 and MS2. The MS data were acquired using the following parameters: full scan MS1 spectral (400–2,000 *m*/*z*) were acquired in the Orbitrap for a maximum ion injection time of auto mode at a resolution of 120,000 and an automatic gain control (AGC) target of standard mode. MS2 spectra were acquired in the Orbitrap mass analyzer at resolution of 30,000 with high-energy collision dissociation of 27% normalized collision energy and AGC target of standard mode with maximum ion injection time of dynamic mode. Previously fragmented ions were excluded for 30 s.

### Analysis of Mass Spectrometry Data

MS/MS spectra were analyzed using the following software analysis protocol with the Uniprot mouse database (release at September 18, 2019) including expected amino acid sequence of Shank3-N (1–549 aa). The reversed sequences of all proteins were appended into the database for calculation of false discovery rate (FDR). ProLuCID (Xu et al., 2006) in Integrated Proteomics Pipeline software (IP2)^1^ was used to identify the peptides, a precursor mass error of 5 ppm, and a fragment ion mass error of 20 ppm. Trypsin was selected as a protease, with two potential missed cleavages. Carbamidomethylation at cysteine was chosen as a static modification. Oxidation at methionine was chosen as variable modifications. The output data files were filtered and sorted to compose the protein list with two and more peptides assignments for spectrum identification at a false positive rate less than 0.01. Finally, all identified tandem mass spectra as Shank3-specific peptides were manually validated.

### Quantification and Statistical Analysis

Values from at least three independent experiments with biological replicates were used for quantification and statistical analyses. All analyses were carried out in a blinded manner. *P* values were calculated by two-tailed Student’s *t*-tests or by one-way or two-way ANOVA with Bonferroni *post hoc* test using GraphPad Prism 5 software. All data are presented as mean ± SEM. ^∗^*P* < 0.05; ^∗∗^*P* < 0.01; ^∗∗∗^*P* < 0.001.

## Results

**FIGURE 1:**
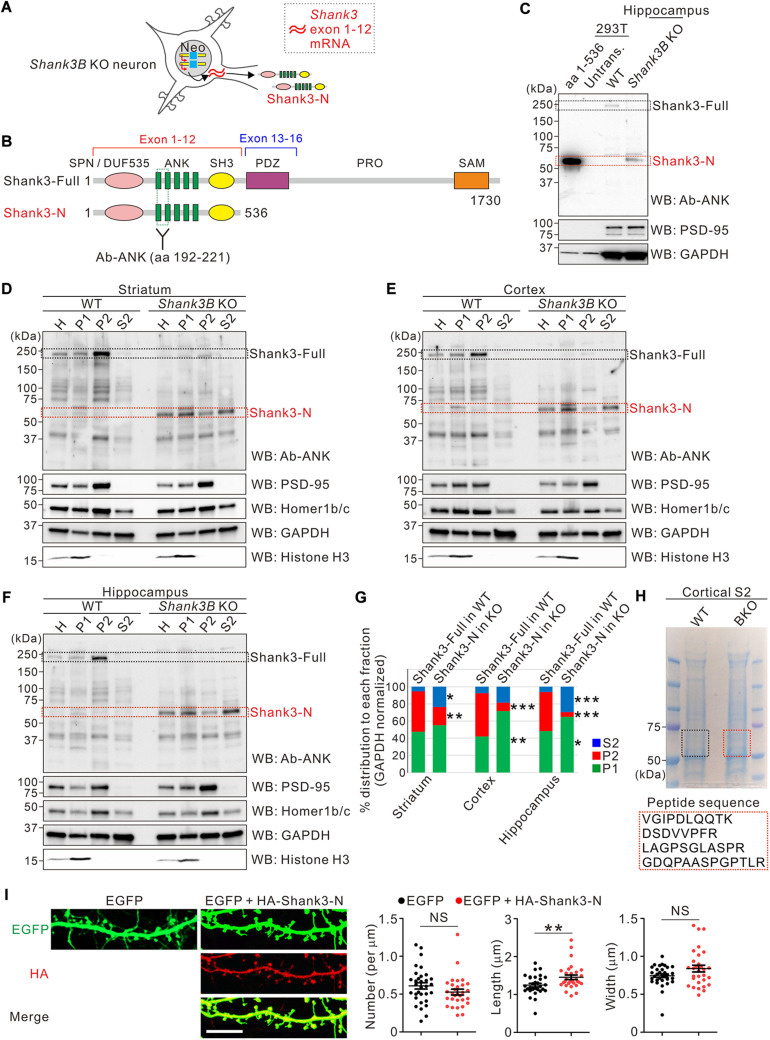
Characterization of the subcellular distribution of Shank3-N protein in *Shank3B* KO brains. **(A)** Schematic diagram showing the expression of *Shank3* exons 1–12 mRNA and Shank3-N protein in *Shank3B* knock-out (KO) neurons. Neo, neomycin resistance cassette. **(B)** Schematic diagram showing the domains of full-length Shank3 (Shank3-Full) and Shank3-N proteins. Location of the epitope for Shank3 N-terminal (Ab-ANK) antibody is indicated. Numbers indicate amino acid residues. ANK, ankyrin repeat domain; DUF535, protein domain of unknown function 535; PDZ, postsynaptic density 95/discs large/zonula occludens 1 domain; PRO, proline-rich region; SAM, sterile alpha motif; SH3, SRC homology 3 domain; SPN, Shank/ProSAP N-terminal domain. **(C)** Western blot (WB) images showing that Shank3-N protein (aa 1–536) expressed in HEK293T cells and the short Shank3 isoform specifically expressed in the *Shank3B* KO hippocampus have similar molecular weights. Untrans, untransfected lysate. **(D–F)** Representative western blot images showing subcellular distributions of Shank3-Full protein in wild-type (WT) brains and Shank3-N protein in *Shank3B* KO brains. PSD-95 and Homer1b/c are synaptic markers, and Histone H3 is a nuclear marker. H, whole homogenate; P1, pellet #1 (nuclear fraction); P2, pellet #2 (crude synaptosomal fraction); S2, supernatant #2 (cytosolic fraction). **(G)** Quantification of relative distributions of Shank3-Full and Shank3-N proteins in each subcellular fraction (striatum, two-way ANOVA, genotype × fraction interaction, *F* = 7.22, *P* = 0.0087, Bonferroni post-tests WT vs. KO, P2 **P < 0.01, S2 *P < 0.05; cortex, interaction *F* = 15.43, *P* = 0.0005, post-tests P1 **P < 0.01, P2 ***P < 0.001; hippocampus interaction *F* = 29.71, *P* < 0.0001, posttests P1 *P < 0.05, P2 ***P < 0.001, S2 ***P < 0.0001; *n* = 4 mice per genotype). **(H)** The S2 fraction samples of WT and *Shank3B* KO mice were loaded on SDS-PAGE gel and were stained with Coomassie blue. The gel pieces between 50 and 75 kDa size markers were cut from the WT and KO lanes, and were further processed for mass spectrometry analyses. The sequences of four Shank3-specific peptides identified only from the KO sample are shown. The mass spectrum peaks are shown in Supplementary Figure S1. **(I)** Representative confocal images of dendritic spines of cultured hippocampal neurons overexpressing EGFP alone or EGFP with HA-Shank3-N. Scale bar, 10 μm. Quantification of the dendritic spine density, length, and width for each condition (*n* = 32 and 30 neurons for EGFP alone and EGFP + HA-Shank3-N, respectively). NS, not significant. Data are presented as mean ± SEM. *P < 0.05, **P < 0.01, ***P < 0.001.

We have previously shown that the mRNA expression of *Shank3* exons 1–12 is higher in *Shank3B* KO brains than in WT brains, as determined by *Shank3* exon-specific qRT-PCR analyses (Jin et al., 2019a). Moreover, we detected an approximately 60 kDa Shank3 protein isoform in multiple brain regions of *Shank3B* KO, but not WT, mice, which may be the product of *Shank3* exons 1–12 in *Shank3B* KO brains (Figure 1A). To better determine the identity of the ∼60 kDa Shank3 protein expressed in *Shank3B* KO brains, we expressed an untagged Shank3 protein in HEK293T cells (Shank3-N, aa 1–536 of 1,730 aa full-length Shank3), corresponding to the Shank3 protein residues encoded by *Shank3* exons 1–12 (Figure 1B). A side-by-side gel running and western blot analysis showed that the Shank3-N protein expressed in HEK293T cells had a similar molecular weight to that of the short Shank3 isoform expressed specifically in *Shank3B* KO brains (Figure 1C), further suggesting that the short Shank3 isoform (now referred to as Shank3-N) was indeed the product of *Shank3* exons 1–12.

Because Shank3-N did not contain the C-terminal sterile alpha motif (SAM) domain of full-length Shank3 (Figure 1B), which is critical for synaptic targeting and multimerization (Boeckers et al., 2005; Baron et al., 2006; Hayashi et al., 2009), we investigated its subcellular distribution in the striatum, cortex, and hippocampus of *Shank3B* KO mice. As expected, full-length Shank3 expressed in the brain regions of WT mice were more highly enriched in the synaptosomal fraction (P2) than in the cytosolic fraction (S2) (Figure 1D–G). In contrast, Shank3-N expressed in *Shank3B* KO brains showed more cytosolic and less synaptosomal distribution than full-length Shank3 in WT mice, suggesting that Shank3-N is not properly targeted to the synaptic compartment. To better determine that the ∼60 kDa protein enriched in the S2 fraction of *Shank3B* KO brains is Shank3-N, we performed mass spectrometry–based analyses on WT and *Shank3B* KO samples (50–75 kDa proteins of the SDS-PAGE gel) (Figure 1H). The analyses identified four Shank3-specific peptides (all matched within 1–536 aa residues of Shank3) from the *Shank3B* KO sample (Supplementary Figure S1). In contrast, no Shank3 peptide was identified from the WT sample.

Based on the aforementioned results, we hypothesized that mislocalized Shank3-N possibly has dominant-negative effects on normal Shank3 functions, such as dendritic spine regulation (Roussignol et al., 2005; Durand et al., 2012), because it contains the N-terminal domains of Shank3 which are involved in interactions with several intracellular signaling regulators (Lilja et al., 2017; Hassani Nia and Kreienkamp, 2018; Yoo Y. E. et al., 2019; Cai et al., 2020; Hassani Nia et al., 2020). To examine this, we co-transfected EGFP and HA-tagged Shank3-N constructs in cultured hippocampal neurons at DIV 7 and analyzed dendritic spines at DIV 20. We observed that Shank3-N proteins were expressed diffusely in cultured neurons, consistent with the results from western blot analyses (Supplementary Figure S2). When compared with control neurons expressing EGFP alone, neurons expressing EGFP and Shank3-N showed a normal dendritic spine density and width, but a significantly increased spine length, suggesting that the overexpression of Shank3-N affects dendritic spine morphology in cultured hippocampal neurons (Figure 1I).

We previously speculated that the increase in the mRNA expression of *Shank3* exons 1–12 and Shank3-N production could be a compensatory response to reduced Shank3 protein levels in *Shank3B* KO brains (Jin et al., 2019a). To validate this hypothesis *in vivo*, we crossed *Shank3B* heterozygous mice (expressing approximately 50% lower levels of Shank3 than that in WT mice) with *EGFP-Shank3* overexpressing transgenic mice (expressing approximately 50% higher levels of Shank3 than that in WT mice) (Han et al., 2013b; Lee et al., 2017b, d) to normalize Shank3 protein levels. The expression pattern of EGFP-Shank3 in transgenic mice is similar to that of endogenous Shank3 because the mice were generated using a bacterial artificial chromosome containing mouse *Shank3* gene and its upstream promoter region (Han et al., 2013b). Moreover, we have previously shown that crossing the two *Shank3* mutant lines not only normalizes Shank3 protein levels but also rescues several behavioral phenotypes, such as locomotor hyperactivity and reduced immobility in the tail-suspension test, observed in *EGFP-Shank3* transgenic mice (Han et al., 2013b; Lee et al., 2018). We reasoned that if an increase in the mRNA expression of *Shank3* exons 1–12 and the production of Shank3-N in *Shank3B* mice was indeed due to a reduction in Shank3, the Shank3-N expression at the mRNA and protein levels would disappear by crossing *Shank3B* heterozygous mice with *EGFP-Shank3* transgenic mice.

**FIGURE 2:**
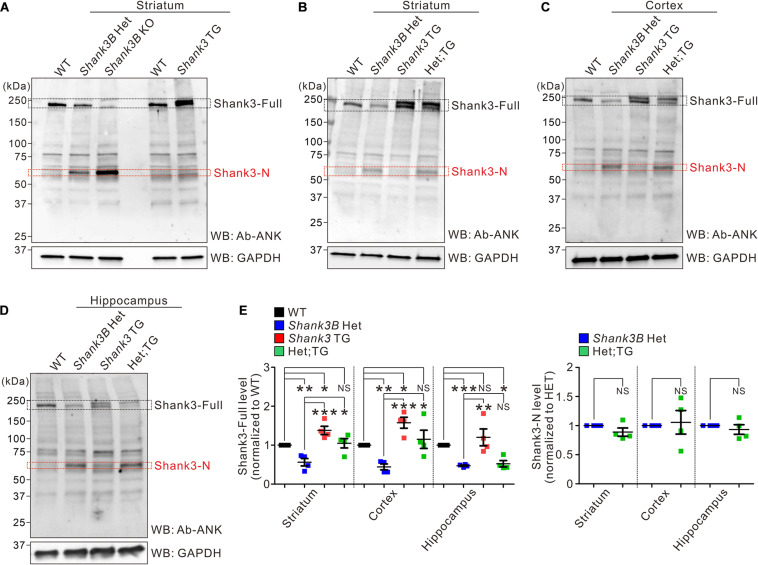
Protein expression of Shank3-N in *Shank3B* KO brains is not due to a reduction of full-length Shank3 proteins. **(A)** Western blot (WB) images showing the protein expression of Shank3-N in the striatum of *Shank3B* heterozygous (HET) and knock-out (KO) mice but not of *EGFP-Shank3* overexpressing transgenic (TG) mice. WT, wild-type. **(B–D)** Representative western blot images showing expression of full-length Shank3 (Shank3-Full) and Shank3-N proteins in the mouse (four different genotypes) striatum, cortex, and hippocampus. Het;TG, double mutant. **(E)** Quantification of expression levels of Shank3-full (left panel) and Shank3-N (right panel) proteins in the brain regions of mice (*n* = 4 mice per genotype). Note that, in the hippocampus, an increase in Shank3-full expression in *Shank3* TG, and thus in Het;TG, mice is milder than those in the striatum and cortex. NS, not significant. Data are presented as mean ± SEM. *P < 0.05, **P < 0.01, ***P < 0.001.

Because the interpretation of qRT-PCR results for *Shank3* exons 1–12 are complicated in *Shank3B*; *EGFP-Shank3* double-mutant mice owing to masking effect by the expression of the *EGFP-Shank3* transgene (Jin et al., 2019a), we instead measured the Shank3-N protein levels by western blot analysis. First, we confirmed that Shank3-N is expressed in the striatum of *Shank3B* heterozygous mice (at a lower level than that in KO mice); however, it was not expressed in *EGFP-Shank3* transgenic mice (Jin et al., 2019a) (Figure 2A). Then, we analyzed the expression levels of Shank3 proteins (both full-length Shank3 and Shank3-N) in the brain regions (striatum, cortex, and hippocampus) of WT, *Shank3B* heterozygous, *EGFP-Shank3* transgenic, and *Shank3B*;*EGFP-Shank3* double-mutant mice (Figures 2B–D). First, as shown previously (Han et al., 2013b), expression levels of full-length Shank3 in the striatum and cortex of double-mutant mice were significantly higher than those of *Shank3B* heterozygous mice and comparable with those of WT mice (Figure 2E, left panel). Surprisingly, however, Shank3-N was still detected in the double-mutant brains at similar levels to those in *Shank3B* heterozygous brains (Figure 2E, right panel). These results suggest that the expression of Shank3-N in *Shank3B* mice could not be a compensatory response to a reduction in Shank3.

The aforementioned results prompted us to investigate genomic changes in the targeted allele of *Shank3B* KO mice, which were generated by replacing exons 13–16 of the *Shank3* gene with the Neo selection cassette (Peca et al., 2011). Several studies have shown that the Neo cassette can affect the expression of neighboring genes in the genome (Pham et al., 1996; Scacheri et al., 2001; Meier et al., 2010; West et al., 2016). Accordingly, we investigated another *Shank3* KO mouse line in parallel, which has a similar *Shank3* exonal deletion as that in *Shank3B* KO mice but without a remaining selection cassette in the targeted allele. In particular, we focused on *Shank3* gKO mice (Yoo T. et al., 2018; Yoo T. et al., 2019), which were generated by deleting exons 14–16 of *Shank3*.

**FIGURE 3:**
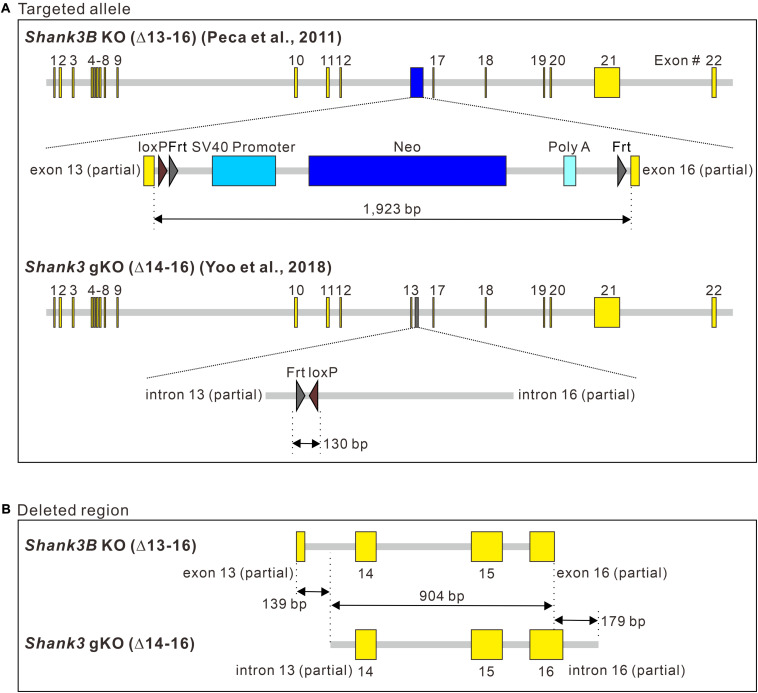
Comparison of the targeted allele sequences of *Shank3B* KO and *Shank3* gKO mice. **(A)** Schematic diagram showing the inserted sequences of the targeted alleles of *Shank3B* knock-out (KO) and *Shank3* gKO mice. bp, base pair. **(B)** Schematic diagram showing side-by-side comparison of the deleted *Shank3* exon and intron sequences in the targeted alleles of *Shank3B* KO and *Shank3* gKO mice. Full sequencing information is provided in Supplementary Material (Supplementary Information 1).

To identify precise genomic changes in the targeted alleles, we sequenced the genomic region around *Shank3* exons 13–16 of the two *Shank3* KO lines (Supplementary Information 1). In terms of the inserted sequence in the targeted allele, an approximately 1.9-kbp Neo cassette sequence containing the promoter, Neo, polyA, loxP, and Frt sites was identified in *Shank3B* KO mice (Figure 3A, upper panel). In *Shank3* gKO mice, only a 130-bp sequence containing Frt and loxP sites was identified (Figure 3A, lower panel). In terms of the deleted sequence from *Shank3*, exons 13–16 were deleted in the targeted allele of *Shank3B* KO mice, but the deletions were partial for exons 13 and 16 (Figure 3B, upper panel). In *Shank3* gKO mice, entire exons 14–16 and partial sequences of the flanking introns (introns 13 and 16) were deleted in the targeted allele (Figure 3B, lower panel). A side-by-side comparison of the deleted sequences in the two *Shank3* KO lines showed that a 904 bp region of *Shank3* gene [from intron 13 (partial) to exon 16 (partial)] was deleted in both lines (Figure 3B). *Shank3B* KO mice had an additional 139 bp deletion in the 5′ side of *Shank3* gene, while *Shank3* gKO mice had an additional 179 bp deletion in the 3′ side of *Shank3* gene. Taken together, these results suggest that the deleted sequences in the two *Shank3* KO lines are largely overlapping, but the inserted sequences in the targeted allele differ substantially (1,923 vs. 130 bp).

**FIGURE 4:**
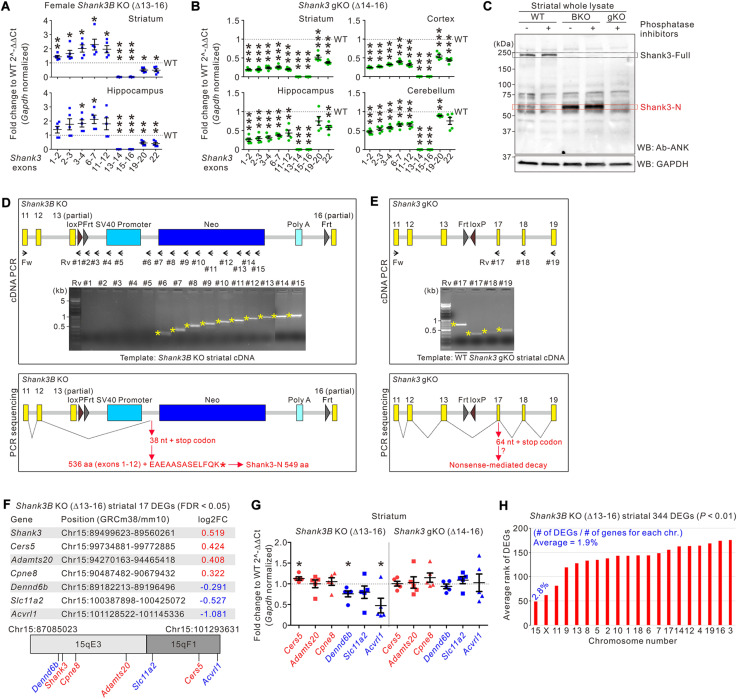
Abnormal expression of *Shank3* exons 1–12 and neighboring genes on mouse chromosome 15 in *Shank3B* KO brains. **(A)** qRT-PCR analysis of exon-specific *Shank3* mRNA levels in the striatum and hippocampus of adult female *Shank3B* knock-out (KO) mice compared with wild-type (WT) littermates (*n* = 5 animals per genotype). **(B)** qRT-PCR analysis of exon-specific *Shank3* mRNA levels in the striatum, cortex, hippocampus, and cerebellum of adult male *Shank3* gKO mice compared with WT littermates (*n* = 5 animals per genotype). **(C)** Western blot (WB) images showing that Shank3-N protein is not expressed in the striatum of *Shank3* gKO mice. The molecular weight of Shank3-N protein in the *Shank3B* KO striatum was not significantly changed by omission of phosphatase inhibitors during protein sample preparation, suggesting that Shank3-N is not highly modified by phosphorylation. **(D)** Schematic diagram showing the locations of PCR primers targeting the Neo cassette of *Shank3B* KO mice. Agarose gel image showing the results of PCR reactions with the primer sets (upper panel). Schematic diagram showing the splicing of *Shank3* exon 12 on the Neo cassette (lower panel). The splicing adds 13 aa residues to Shank3 encoded by exons 1–12 to produce Shank3-N protein (549 aa residues). Fw, forward; Rv, reverse. **(E)** Schematic diagrams showing the locations of PCR primers (upper panel) and the splicing of *Shank3* exons in *Shank3* gKO mice (lower panel). The splicing causes frameshift and generates a premature stop codon, which may lead to nonsense-mediated decay of the mRNA. **(F)** Summary of the seven differentially expressed genes (DEGs) in the *Shank3B* KO striatum on mouse chromosome 15. Their genomic positions and fold change (FC) values are indicated. **(G)** qRT-PCR analysis of the six DEGs on mouse chromosome 15 in the striatum of *Shank3B* KO and *Shank3* gKO mice compared with respective WT controls (*n* = 5 animals per genotype). **(H)** Chromosomal distribution (normalized to total number of genes for each chromosome) and rank values of 344 DEGs in the *Shank3B* KO striatum. chr, chromosome. Data are presented as mean ± SEM. *P < 0.05, **P < 0.01, ***P < 0.001.

If the Neo cassette inserted in the targeted allele, rather than a Shank3 protein reduction (as shown in Figure 2), in *Shank3B* KO mice is involved in the increase in the mRNA expression of *Shank3* exons 1–12, the increase should not be observed in the brain regions of *Shank3* gKO mice. First, we confirmed that the mRNA expression levels of *Shank3* exons 1–12 increased in the striatum and hippocampus of adult female *Shank3B* KO mice (Figure 4A), suggesting that this increase occurs in *Shank3B* KO mice, regardless of age (at both the juvenile and adult stages) and sex (Jin et al., 2019a). Next, we applied the same *Shank3* exon-specific qRT-PCR analysis to the striatum, cortex, hippocampus, and cerebellum of *Shank3* gKO mice. Notably, in all four brain regions of *Shank3* gKO mice, mRNA expression levels of all *Shank3* exons 1–22 were lower than those in WT mice (Figure 4B). Consistent with the mRNA analysis, even with the loss of full-length Shank3 proteins as in *Shank3B* KO mice, the ∼60 kDa Shank3-N protein was not detected in the *Shank3* gKO striatum (Figure 4C). This result further suggests that expression of Shank3-N in *Shank3B* KO mice was not due to a reduction of full-length Shank3 protein (Figure 2). Taken together, these results demonstrate a significant contrast in mRNA and protein expression patterns of *Shank3* exons 1–12 between *Shank3B* KO and *Shank3* gKO brains.

To better understand the *Shank3* mRNA and protein products in *Shank3B* KO and *Shank3* gKO brains, we performed PCR with the striatal cDNA of *Shank3B* KO and *Shank3* gKO mice using a forward primer targeting exon 11 and reverse primers targeting different regions of the targeted alleles (Figures 4D,E and Supplementary Information 2). In the case of *Shank3B* KO mice, PCR products were observed when the forward primer was combined with reverse primers targeting after the SV40 promoter sequence, suggesting that Shank-N mRNA may end with the Neo and polyA sequence of the cassette (Figure 4D, upper panel). Indeed, direct sequencing of the PCR products showed that *Shank3* exon 12, skipping the partial exon 13, spliced on the region between SV40 promoter and Neo (Figure 4D, lower panel and Supplementary Information 3). This splicing event may add 13 aa residues (EAEAASASELFQK) to the C terminus of 536 aa residues encoded by *Shank3* exons 1–12 to produce Shank3-N protein (total 549 aa residues). In the case of *Shank3* gKO mice, similar PCR and sequencing approaches showed that *Shank3* exon 13 spliced on exon 17, which induces frameshift and a premature stop codon (Figure 4E and Supplementary Information 3). We speculate that this *Shank3* mRNA in *Shank3* gKO brains may undergo nonsense-mediated decay because its mRNA levels were very low (Figure 4B) and because expected proteins from the mRNA were not detected (Figure 4C).

The Neo cassette can have long-range off-target effects on the expression of neighboring genes (Pham et al., 1996). Therefore, we investigated expression changes in genes neighboring *Shank3* on mouse chromosome 15 of *Shank3B* KO mice. Notably, in our previous RNA-sequencing analysis of the striatum of *Shank3B* KO mice (Lee et al., 2019a), we found that 7 out of 17 (approximately 41%, including *Shank3*) differentially expressed genes (DEGs) were on mouse chromosome 15 (Figure 4F). Other than *Shank3*, three DEGs (*Cers5*, *Adamts20*, and *Cpne8*) were upregulated, and the other three DEGs (*Dennd6b*, *Slc11a2*, and *Acvrl1*) were downregulated in the striatum of *Shank3B* KO mice. To validate these results, we performed qRT-PCR analyses of the six genes in the striata of both *Shank3B* KO and *Shank3* gKO mice, together with their respective WT littermates. In the *Shank3B* KO striatum, expression levels of *Cers5* were significantly higher and levels of *Dennd6b* and *Acvrl1* were significantly lower than those in the WT striatum (Figure 4G). In contrast, however, none of the six genes showed significant expression changes in *Shank3* gKO mice, suggesting that changes in the expression of genes neighboring *Shank3* occur specifically in *Shank3B* KO mice.

To further understand the overall gene expression changes in the *Shank3B* KO striatum, we expanded the DEG list from 17 to 344 by applying a less stringent statistical criterion (from FDR < 0.05 to *P* < 0.01). Notably, when normalized to the total number of genes on chromosome 15 (673 genes), 2.8% of genes (19 out of 673) were identified in the list of 334 DEGs, which was the highest percentage among the 20 mouse chromosomes (average 1.9% of genes) (Figure 4H). Moreover, when the 344 DEGs were ranked based on *P* values, the average rank value of the 19 DEGs on chromosome 15 was the lowest (i.e., the DEGs were top-ranked) compared with average ranks of DEGs on other chromosomes (Figure 4H, red bars). These results suggest that, in the striatum of *Shank3B* KO mice, gene expression changes were overrepresented on chromosome 15.

## Discussion

In this study, we showed that Shank3-N protein expressed in *Shank3B* KO brains was less efficiently targeted to the synaptic compartment than full-length Shank3, possibly because it has only the N-terminal [Shank/ProSAP N-terminal (SPN), ankyrin repeat (ANK), and SRC homology 3 (SH3)] domains but not the C-terminal SAM domain of full-length Shank3. The N-terminal domains of Shank3 interact with several proteins, such as Ras and Rap G-proteins (Lilja et al., 2017), α-fordin (Bockers et al., 2001), Densin-180 (Quitsch et al., 2005), Ca_v_1.3 L-type calcium channel (Zhang et al., 2005), and Sharpin (Lim et al., 2001), which are involved in the regulation of diverse neuronal functions. We carefully speculate that Shank3-N protein may possibly interact with some of the aforementioned proteins, and thereby disturbing their proper synaptic localization and function. Moreover, a recent study has identified nuclear localization signals in the N-terminal part of Shank3 (between the ANK and SH3 domains) (Hassani Nia et al., 2020). Supporting its functional importance, several types of missense mutations in the N-terminal part of Shank3 have been identified in patients with ASDs (Hassani Nia and Kreienkamp, 2018). Therefore, Shank3-N expression, together with loss of full-length Shank3, may, at least partially, contribute to the neuronal phenotypes observed in *Shank3B* KO mice. This is further supported by our observation that Shank3-N overexpression changed dendritic spine morphology in cultured hippocampal neurons. However, this result was obtained in WT neurons expressing endogenous full-length Shank3, and thus may not fully reflect Shank3-N function in *Shank3B* KO neurons.

We provided several lines of evidence suggesting that the Neo cassette in the targeted allele of *Shank3B* KO mice has potential off-target effects on the expression of *Shank3* and its neighboring genes on mouse chromosome 15. First, we excluded the possibility that Shank3-N expression in *Shank3B* mice was a compensatory response to a reduction of full-length Shank3. Specifically, Shank3-N was still detected even after normalizing the level of full-length Shank3 by crossing *Shank3B* heterozygous mice with *Shank3* overexpressing transgenic mice. In addition, Shank3-N was not detected in the brains of *Shank3* gKO mice, despite the loss of full-length Shank3 as in *Shank3B* KO mice.

Second, we compared the targeted allele sequences of *Shank3B* KO and *Shank3* gKO mice. This revealed that deleted sequences of *Shank3* gene in the two KO lines were largely overlapping, while retained sequences of selection markers differed substantially, suggesting that the latter sequences may be more important determinants of gene expression differences between the two KO lines. Moreover, no gene regulatory element was identified around the genomic region of *Shank3* exons 13–16 (Supplementary Figure S3), suggesting that integration of the exogenous Neo cassette, rather than the removal of the *Shank3* sequence, likely resulted in the abnormal gene expression in *Shank3B* KO brains. By performing PCR with different primer sets followed by direct sequencing, we predicted the *Shank3* mRNA and protein products in *Shank3B* KO and *Shank3* gKO mice. We carefully speculate that the *Shank3* mRNA in *Shank3* gKO, but not *Shank3B* KO, brains may undergo nonsense-mediated decay. It is not immediately clear how Shank3-N mRNA in *Shank3B* KO mice escapes from nonsense-mediated decay. Nevertheless, the escape alone may not be enough to fully explain the expression of Shank3-N mRNA in *Shank3B* KO mice because its levels are upregulated compared with *Shank3* transcripts in WT mice.

Third, in addition to *Shank3* exons 1–12, neighboring genes on mouse chromosome 15, such as *Cers5*, *Dennd6b*, and *Acvrl1*, showed altered expression levels in the striatum of *Shank3B* KO mice, but not in *Shank3* gKO mice. Furthermore, genes with expression changes in the striatum of *Shank3B* KO mice were disproportionately clustered on chromosome 15 (41% of DEGs). When we re-analyzed the RNA-sequencing data on the prefrontal cortex of another *Shank3* KO line (*Shank3ΔC* carrying deletion of exon 21) (Qin et al., 2018), only 2 out of 45 DEGs (including *Shank3* itself) were on chromosome 15 (Jin et al., 2018), further indicating that gene expression changes in *Shank3B* KO brains are unusual. Nevertheless, whether these effects were solely due to the Neo cassette in the targeted allele of *Shank3B* KO mice and how the Neo cassette interacted with *Shank3* exons 1–12 and its neighboring genes are not immediately clear. Moreover, it cannot be excluded that Shank3-N protein may secondarily affect gene expression in *Shank3B* KO brains by modulating intracellular signals. Notably, we found that the Neo cassette of *Shank3B* KO mice is flanked by Frt sites (Figure 3A). Therefore, it will be an interesting topic for future studies to determine whether the Neo cassette can be removed using the Frt sites and whether expression changes of *Shank3* exons 1–12 and neighboring genes in *Shank3B* KO mice can be attenuated by removing the Neo cassette. Moreover, it will be another interesting topic to investigate whether those genes (*Cers5*, *Dennd6b*, and *Acvrl1*) on chromosome 15 with altered mRNA expression levels in *Shank3B* KO mice have any functional effects. Validation of their protein level changes (ceramide synthase 5, DENN domain containing 6B, and activin A receptor like type 1 which are encoded by *Cers5*, *Dennd6b*, and *Acvrl1*, respectively) in *Shank3B* KO brains compared with WT brains is a prerequisite for addressing this issue.

In conclusion, our results emphasize the need for the careful characterization and interpretation of gene expression changes in *Shank3B* KO mice. More broadly, it is possible that our findings can be extended to other *Shank3*, and *Shank1* and *Shank2*, KO lines (Eltokhi et al., 2018) with selection markers retained in the targeted alleles. Potential off-target effects of selection markers, together with Shank3 isoform diversity (Wang et al., 2014) and compensatory changes in *Shank3* expression (Qin et al., 2018; Jin et al., 2019a), may collectively contribute to phenotypic heterogeneity among different *Shank3* KO lines, validation of which requires further investigations.

## Data Availability Statement

All raw MS and supporting data files from this study have been deposited to the repository MassIVE with identifier PDX022828, https://massive.ucsd.edu/ProteoSAFe/dataset.jsp?task=ac7adebf965441f298dbf993e9a28058.

## Ethics Statement

The animal study was reviewed and approved by the Committee on Animal Research at Korea University College of Medicine.

## Author Contributions

CJ, TY, JR, Y-EY, RM, YZ, HRK, YK, HS, GB, SP, and KH designed and performed the experiments. HKa, JK, S-KK, WS, HKi, EK, and KH analyzed and interpreted the data. KH wrote the manuscript. All authors read and approved the article.

## Conflict of Interest

The authors declare that the research was conducted in the absence of any commercial or financial relationships that could be construed as a potential conflict of interest.

## Footnotes

1 www.integratedproteomics.com
