# Supplementary material for: The Neomycin Resistance Cassette in the Targeted Allele of Shank3B Knock-Out Mice Has Potential Off-Target Effects to Produce an Unusual Shank3 Isoform
Source: Front Mol Neurosci. 2021 Jan 11;13:614435. doi: 10.3389/fnmol.2020.614435 (PMC7831789; doi:10.3389/fnmol.2020.614435)
Supplement: Supplementary file 1 [file Data_Sheet_1.docx]

**Supplementary material**

**The neomycin resistance cassette in the targeted allele of *Shank3B* knock-out mice has potential off-target effects to produce an unusual Shank3 isoform**

Chunmei Jin, Hyojin Kang, Taesun Yoo, Jae Ryun Ryu, Ye-Eun Yoo, Ruiying Ma, Yinhua Zhang, Hyae Rim Kang, Yoonhee Kim, Hyunyoung Seong, Geul Bang, Sangwoo Park, Seok-Kyu Kwon, Woong Sun, Hyunkyung Kim, Jin Young Kim, Eunjoon Kim, and Kihoon Han

**Figure S1.** Four Shank3-specific peptides identified from the S2 fraction sample of *Shank3B* KO mice.

**Figure S2.** Two representative images of cultured neurons expressing EGFP and HA-Shank3-N proteins.

**Figure S3.** Identification of gene regulatory elements in mouse *Shank3* gene.

**Supplementary Information 1.** Sequencing results of the targeted alleles of *Shank3B* KO and *Shank3* gKO mice.

**Supplementary Information 2.** PCR primers for Figures 4D and E

**Supplementary Information 3.** Sequencing results for Figures 4D and E


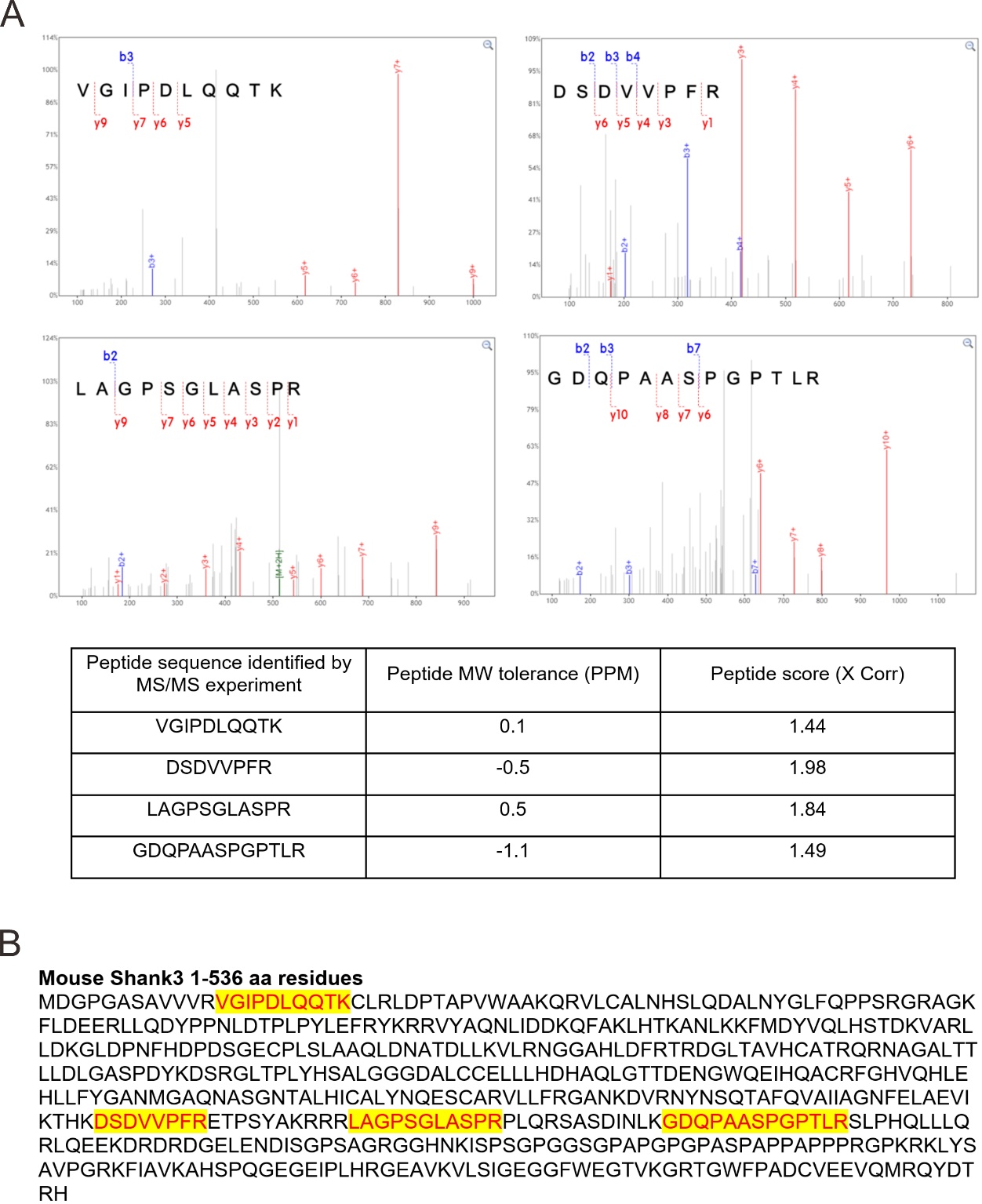


**Figure S1. Four Shank3-specific peptides identified from the S2 fraction sample of *Shank3B* KO mice.** **(A)** The mass spectrum peaks for each Shank3 peptides. **(B)** The locations of four peptides within mouse Shank3 1-536 aa residues.


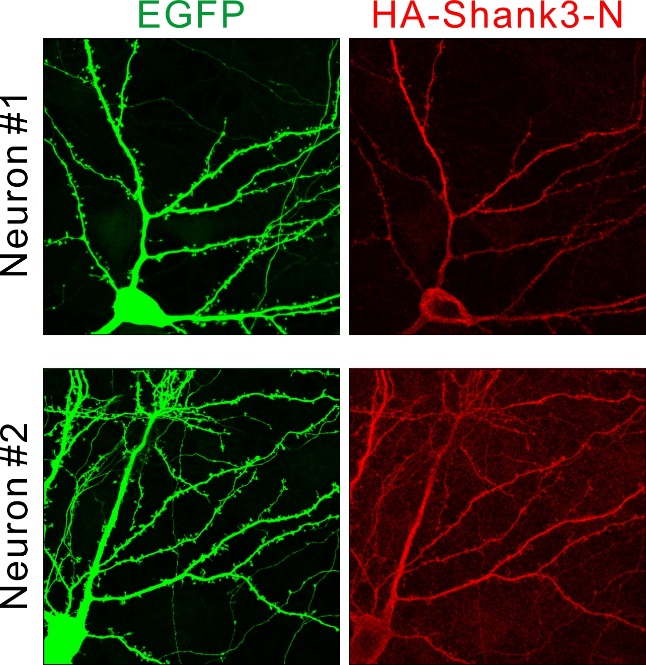


**Figure S2. Two representative images of cultured neurons expressing EGFP and HA-Shank3-N proteins.** Cultured hippocampal neurons at days in vitro 20 were fixed and immunostained with anti-GFP and anti-HA antibodies.

**
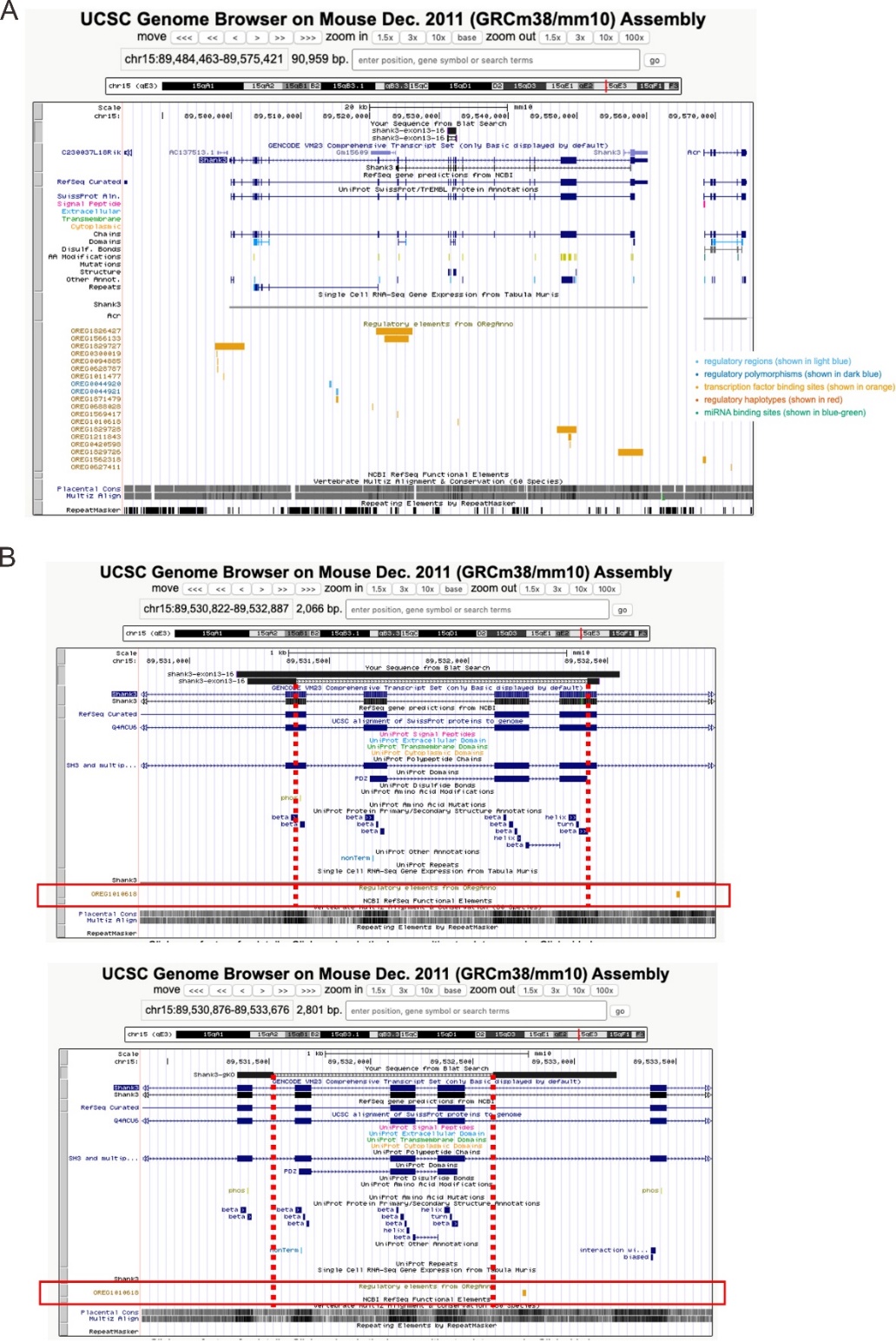
**

**Figure S3. Identification of gene regulatory elements in mouse *Shank3* gene. (A)** Identification of gene regulatory elements (green box) in mouse *Shank3* gene using the Open Regulatory Annotation database (ORegAnno) tool ([Lesurf et al., 2016](#_ENREF_1)) of the genome browser. **(B)** No gene regulatory element was identified in the deleted regions (between dotted lines) of *Shank3* gene in *Shank3B* KO (upper panel) and *Shank3* gKO (lower panel) mice.

**Reference** Lesurf et al., (2016). ORegAnno 3.0: a community-driven resource for curated regulatory annotation. *Nucleic Acids Res* 44**,** D126-132.

**Supplementary Information 1. Sequencing results of the targeted alleles of *Shank3B* KO and *Shank3* gKO mice.**

**1. Targeted allele of *Shank3B* KO mice**

*Shank3* exon 13 loxP Frt SV40 Promoter Neo Poly A Frt *Shank3* exon 16 1,923 bp

gatttgcccccacccccatccccactcacatcgaggcccagagagtgtgggggcagagactgatcagcgcagttgggggaaattcagctaggtggagggtcacgactgtttcttagcctttgatgctccccgcagAAACCAGAGAGGACCGGACGAAGCGTCTCTTCCGCCACTACAGGCGCGCCTAGTCGACATAACTTCGTATAGCATACATTATACGAAGTTATAGCGCGAAGTTCCTATTCTCTAGAAAGTATAGGAACTTCAAGCTTAGGTGGCACTTTTCGGGGAAATGTGCGCGGAACCCCTATTTGTTTATTTTTCTAAATACATTCAAATATGTATCCGCTCATGAGACAATAACCCTGATAAATGCTTCAATAATATTGAAAAAGGAAGAGTCCTGAGGCGGAAAGAACCAGCTGTGGAATGTGTGTCAGTTAGGGTGTGGAAAGTCCCCAGGCTCCCCAGCAGGCAGAAGTATGCAAAGCATGCATCTCAATTAGTCAGCAACCAGGTGTGGAAAGTCCCCAGGCTCCCCAGCAGGCAGAAGTATGCAAAGCATGCATCTCAATTAGTCAGCAACCATAGTCCCGCCCCTAACTCCGCCCATCCCGCCCCTAACTCCGCCCAGTTCCGCCCATTCTCCGCCCCATGGCTGACTAATTTTTTTTATTTATGCAGAGGCCGAGGCCGCCTCGGCCTCTGAGCTATTCCAGAAGTAGTGAGGAGGCTTTTTTGGAGGCCTAGGCTTTTGCAAAGATCGATCAAGAGACAGGATGAGGATCGTTTCGCATGATTGAACAAGATGGATTGCACGCAGGTTCTCCGGCCGCTTGGGTGGAGAGGCTATTCGGCTATGACTGGGCACAACAGACAATCGGCTGCTCTGATGCCGCCGTGTTCCGGCTGTCAGCGCAGGGGCGCCCGGTTCTTTTTGTCAAGACCGACCTGTCCGGTGCCCTGAATGAACTGCAAGACGAGGCAGCGCGGCTATCGTGGCTGGCCACGACGGGCGTTCCTTGCGCAGCTGTGCTCGACGTTGTCACTGAAGCGGGAAGGGACTGGCTGCTATTGGGCGAAGTGCCGGGGCAGGATCTCCTGTCATCTCACCTTGCTCCTGCCGAGAAAGTATCCATCATGGCTGATGCAATGCGGCGGCTGCATACGCTTGATCCGGCTACCTGCCCATTCGACCACCAAGCGAAACATCGCATCGAGCGAGCACGTACTCGGATGGAAGCCGGTCTTGTCGATCAGGATGATCTGGACGAAGAGCATCAGGGGCTCGCGCCAGCCGAACTGTTCGCCAGGCTCAAGGCGAGCATGCCCGACGGCGAGGATCTCGTCGTGACCCATGGCGATGCCTGCTTGCCGAATATCATGGTGGAAAATGGCCGCTTTTCTGGATTCATCGACTGTGGCCGGCTGGGTGTGGCGGACCGCTATCAGGACATAGCGTTGGCTACCCGTGATATTGCTGAAGAGCTTGGCGGCGAATGGGCTGACCGCTTCCTCGTGCTTTACGGTATCGCCGCTCCCGATTCGCAGCGCATCGCCTTCTATCGCCTTCTTGACGAGTTCTTCTGAGCGGGACTCTGGGGTTCGAAATGACCGACCAAGCGACGCCCAACCTGCCATCACGAGATTTCGATTCCACCGCCGCCTTCTATGAAAGGTTGGGCTTCGGAATCGTTTTCCGGGACGCCGGCTGGATGATCCTCCAGCGCGGGGATCTCATGCTGGAGTTCTTCGCCCACCCTAGGGGGAGGCTAACTGAAACACGGAAGGAGACAATACCGGAAGGAACCCGCGCTATGACGGCAATAAAAAGACAGAATAAAACGCACGGTGTTGGGTCGTTTGTTCATAAACGCGGGGTTCGGTCCCAGGGCTGGCACTCTGTCGATACCCCACCGAGACCCCATTGGGGCCAATACGCCCGCGTTACTTCCTTTTCCCCACCCCACCCCCCAAGTTCGGGTGAAGGCCCAGGGCTCGCAGCCAACGTCGGGGCGGCAGGCCCTGCCAGGATCCGAAGTTCCTATTCTCTAGAAAGTATAGGAACTTCACCGGTTAATTAAGGGCTCGATCGACCGGAGGAAACCCGAGGAGGATGGTGCTCGGCGCAGAGgtgagggt

**2. Targeted allele of *Shank3* gKO mice**

*Shank3* exon 13 *Shank3* intron 13 Frt loxP *Shank3* intron 16 130 bp

AAACCAGAGAGGACCGGACGAAGCGTCTCTTCCGCCACTACACTGTGGGTTCCTATGACAGCCTCACTTCACACAGgtactggcaggggtgtggacacgcagggacagtagcagggcctagggtgtccagtgaaagggggaagtcagggtatggggagtgggtggctagcttcctctgcctAATATTATGCATAGCGCTGAAGTTCCTATTCTCTAGAAAGTATAGGAACTTCATCAGTCAGGTACATAATATAACTTCGTATAATGTATGCTATACGAAGTTATACGCGTAGATCTCATATGAAGCTTAGatgcctgacctctgaccaggcctctgatccctgatcctagattacccctcctcagttccctctccccttctcctgtgaatccccccagccctggtgtggcagagccctcctttccgacgccagcagctgcctggaggccgggttgtcatggcaaccgtgaggttgacgctgctgctgctgctgcttattgtgcagggaagggggaggcggcacctgagggaagaggaaaagcgtcttctccccgcgccgacgccaaagacgcggcaccgcctgggcctaagaggctttgccgccggcccagcccaggtgtgccgcctgccgggccacactttggtcttacaaagagcagtggtacctgggtctgggtgctttaggcggcaggaggtagcctggcaggagatctacccagcccccatgggtggtgcccactcactggggccttggagcccgcagggtggatcctcagagcagaaccagccagcatcctagcctttgctggtgcttttattaccatttgaggccttggtcctgggagtctgaagagccctaggggcacaactgccctggctccactgctccctgagtgcctcacgccatgaagaa

**3. Deleted sequence in *Shank3B* KO mice (in red)**

*Shank3* exons 13-16 *Shank3* introns 13-15

AAACCAGAGAGGACCGGACGAAGCGTCTCTTCCGCCACTACACTGTGGGTTCCTATGACAGCCTCACTTCACACAGgtactggcaggggtgtggacacgcagggacagtagcagggcctagggtgtccagtgaaagggggaagtcagggtatggggagtgggtggctagcttcctctgcctttgtcattctgtgtggtctggtgtgtatgggaccctgtgggttcagcctgactgccaggctccctgcagacctcccagctcaaccttgctttcctttgccagCGATTATGTCATCGATGATAAGGTGGCTATCCTGCAGAAAAGGGACCATGAGGGGTTTGGCTTTGTTCTCCGGGGAGCCAAAGgtaaagggtgtgaatttggggggtatcgcattgctgtcaatgtcttctgtggcctgcctctgccccttttccccttttaaagccctctgcttcccccccaccccccgtggttatatgtcttggtttggaacatactggggtacaagacagtggatccctctccttttagcctttcatttccttgtggggatctctgctcctggtgaaacagagatctttttgctgagggtaaatgtggaagttcacagagggatgtacagtcatttggatatccctacctgctgcctctcccaccccattgctatgccttagtagtcaggtggcttcttggggctccccgacatgatctatgcttgtgaatgccttttatcttgctctccctgcagCAGAGACCCCCATTGAGGAGTTTACACCCACACCTGCCTTCCCTGCACTCCAATACCTTGAGTCTGTAGATGTGGAAGGTGTGGCCTGGAGGGCTGGACTTCGAACTGGGGACTTCCTCATTGAGgtgaggcccgtcccgaccctgtccagcaggagggggcgagagcactactaactgccacctgaagaggggacagctggcctctgactacctagctgcttgtctatcccagGTGAACGGAGTGAATGTCGTGAAGGTTGGACACAAGCAAGTGGTGGGTCTCATCCGTCAGGGTGGCAACCGCCTGGTCATGAAGGTTGTGTCTGTGACCAGGAAACCCGAGGAGGATGGTGCTCGGCGCAGAG

**4. Deleted sequence in *Shank3* gKO mice (in red)**

*Shank3* exons 13-16 *Shank3* introns 13-16

AAACCAGAGAGGACCGGACGAAGCGTCTCTTCCGCCACTACACTGTGGGTTCCTATGACAGCCTCACTTCACACAGgtactggcaggggtgtggacacgcagggacagtagcagggcctagggtgtccagtgaaagggggaagtcagggtatggggagtgggtggctagcttcctctgcctttgtcattctgtgtggtctggtgtgtatgggaccctgtgggttcagcctgactgccaggctccctgcagacctcccagctcaaccttgctttcctttgccagCGATTATGTCATCGATGATAAGGTGGCTATCCTGCAGAAAAGGGACCATGAGGGGTTTGGCTTTGTTCTCCGGGGAGCCAAAGgtaaagggtgtgaatttggggggtatcgcattgctgtcaatgtcttctgtggcctgcctctgccccttttccccttttaaagccctctgcttcccccccaccccccgtggttatatgtcttggtttggaacatactggggtacaagacagtggatccctctccttttagcctttcatttccttgtggggatctctgctcctggtgaaacagagatctttttgctgagggtaaatgtggaagttcacagagggatgtacagtcatttggatatccctacctgctgcctctcccaccccattgctatgccttagtagtcaggtggcttcttggggctccccgacatgatctatgcttgtgaatgccttttatcttgctctccctgcagCAGAGACCCCCATTGAGGAGTTTACACCCACACCTGCCTTCCCTGCACTCCAATACCTTGAGTCTGTAGATGTGGAAGGTGTGGCCTGGAGGGCTGGACTTCGAACTGGGGACTTCCTCATTGAGgtgaggcccgtcccgaccctgtccagcaggagggggcgagagcactactaactgccacctgaagaggggacagctggcctctgactacctagctgcttgtctatcccagGTGAACGGAGTGAATGTCGTGAAGGTTGGACACAAGCAAGTGGTGGGTCTCATCCGTCAGGGTGGCAACCGCCTGGTCATGAAGGTTGTGTCTGTGACCAGGAAACCCGAGGAGGATGGTGCTCGGCGCAGAGgtgagggttctgatggctgggctttcagacctttgacttcaagcccacattcctcttccatttctgatcctacacagctgacatcacattgctgcctgagatgagataaaatgcccttatttgctgcacaggtccttggtgttagatgcctgacctctgaccaggcctctgatccctgatcctagattacccctcctcagttccctctccccttctcctgtgaatccccccagccctggtgtggcagagccctcctttccgacgccagcagctgcctggaggccgggttgtcatggcaaccgtgaggttgacgctgctgctgctgctgcttattgtgcagggaagggggaggcggcacctgagggaagaggaaaagcgtcttctccccgcgccgacgccaaagacgcggcaccgcctgggcctaagaggctttgccgccggcccagcccaggtgtgccgcctgccgggccacactttggtcttacaaagagcagtggtacctgggtctgggtgctttaggcggcaggaggtagcctggcaggagatctacccagcccccatgggtggtgcccactcactggggccttggagcccgcagggtggatcctcagagcagaaccagccagcatcctagcctttgctggtgcttttattaccatttgaggccttggtcctgggagtctgaagagccctaggggcacaactgccctggctccactgctccctgagtgcctcacgccatgaagaagtttgcatctagccgcagcctgaacaagatcttggcacagtgtgactcctcttccagagagtacgaggaggtccaggcagtggagcgcaagtggcatttgcacctggccactccgcgccgcctgctgctggaccggagggccaaggcctccctcttctttgcag

**Supplementary Information 2. PCR primers for Figures 4D and E**

Fw: 5’ GCCCGAAGCGGAAACTTTA 3’

Rv #1: 5’ TTCGCGCTATAACTTCGTATAATGTA 3’

Rv #2: 5’ AAAAGTGCCACCTAAGCTTGAA 3’

Rv #3: 5’ GCGGATACATATTTGAATGTATTTAG 3’

Rv #4: 5’ ACACATTCCACAGCTGGTTC 3’

Rv #5: 5’ GACTTTCCACACCTGGTTGC 3’

Rv #6: 5’ AGCCTCCTCACTACTTCTGGAAT 3’

Rv #7: 5’ GGAGAACCTGCGTGCAAT 3’

Rv #8: 5’ CGGTCTTGACAAAAAGAACC 3’

Rv #9: 5’ CACAGCTGCGCAAGGAAC 3’

Rv #10: 5’ ATACTTTCTCGGCAGGAGCA 3’

Rv #11: 5’ GAGTACGTGCTCGCTCGAT 3’

Rv #12: 5’ CATGGGTCACGACGAGAT 3’

Rv #13: 5’ TAGCCAACGCTATGTCCTGATAG 3’

Rv #14: 5’ GCGATACCGTAAAGCACGAG 3’

Rv #15: 5’ AGAACTCGTCAAGAAGGCGATA 3’

Rv #17: 5’ CGTCATGGACTTGGACCG 3’

Rv #18: 5’ TCTCCGAATGGAAGCAAG 3’

Rv #19: 5’ ATGTCCGGCCTCAGTGTC 3’

**1. *Shank3B* KO mice**


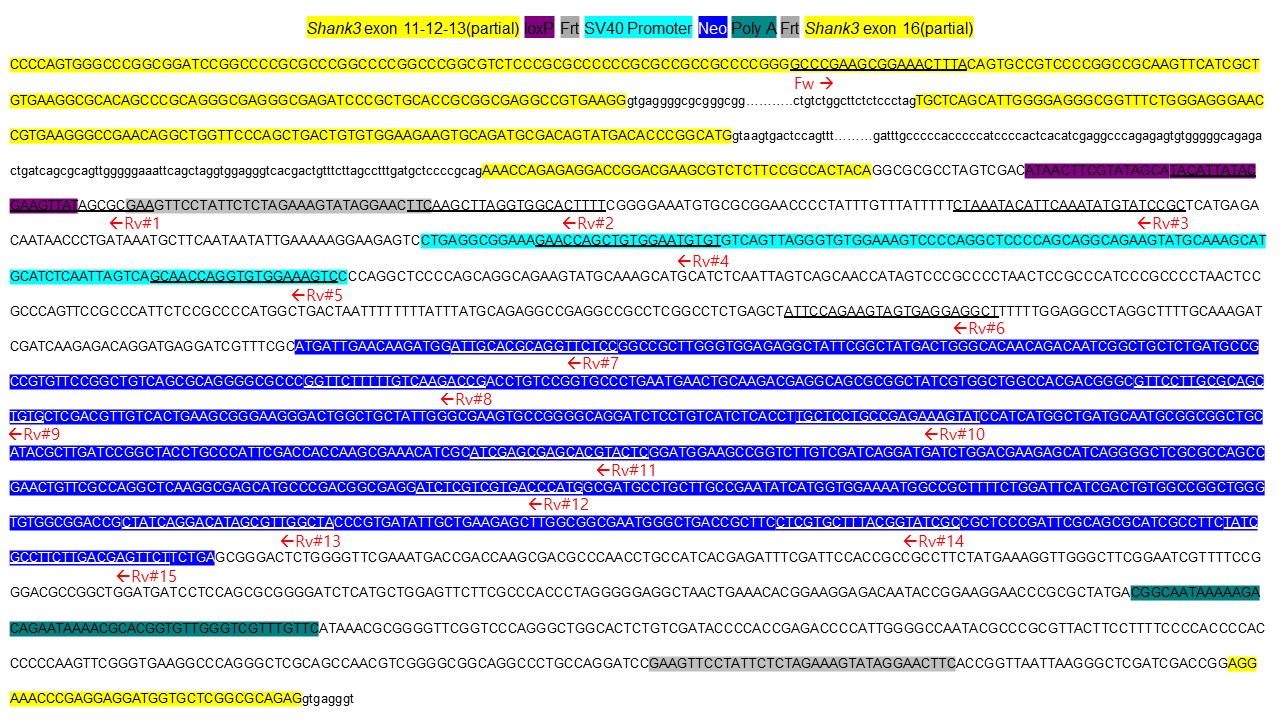


**2. *Shank3* gKO mice**


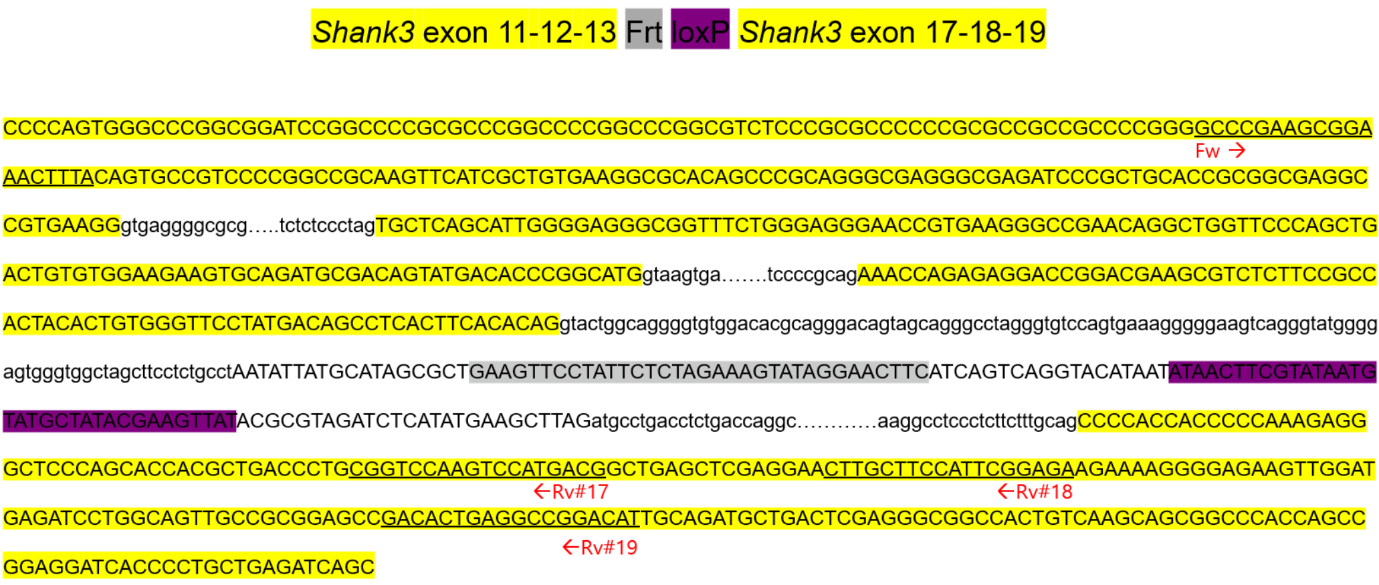


**Supplementary Information 3. Sequencing results for Figures 4D and E**

**1. *Shank3B* KO mice**

**1) PCR product sequencing**

Shank3 exon12 cassette sequence between SV40 promoter and Neo (partial) Neo

TGCTCAGCATTGGGGAGGGCGGTTTCTGGGAGGGAACCGTGAAGGGCCGAACAGGCTGGTTCCCAGCTGACTGTGTGGAAGAAGTGCAGATGCGACAGTATGACACCCGGCATGAGGCCGAGGCCGCCTCGGCCTCTGAGCTATTCCAGAAGTAGTGAGGAGGCTTTTTTGGAGGCCTAGGCTTTTGCAAAGATCGATCAAGAGACAGGATGAGGATCGTTTCGCATGATTGAACAAGATGGATTGCACGCAGGTTCTCCGGCCGCTTGGGTGGAGAGGCTATTCGGCTATGACTGGGCACAACAGACAATCGGCTGCTCTGATGCCGCCGTGTTCCGGCTGTCAGCGCAGGGGCGCCCGGTTCTTTTTGTCAAGAC...

**2) In-frame codon (stop codon)**

TGCTCAGCATTGGGGAGGGCGGTTTCTGGGAGGGAACCGTGAAGGGCCGAACAGGCTGGTTCCCAGCTGACTGTGTGGAAGAAGTGCAGATGCGACAGTATGACACCCGGCATGAGGCCGAGGCCGCCTCGGCCTCTGAGCTATTCCAGAAG**TAG**TGAGGAGGCTTTTTTGGAGGCCTAGGCTTTTGCAAAGATCGATCAAGAGACAGGATGAGGATCGTTTCGCATGATTGAACAAGATGGATTGCACGCAGGTTCTCCGGCCGCTTGGGTGGAGAGGCTATTCGGCTATGACTGGGCACAACAGACAATCGGCTGCTCTGATGCCGCCGTGTTCCGGCTGTCAGCGCAGGGGCGCCCGGTTCTTTTTGTCAAGAC

**3) Protein translation**

exon 12 additional 13 residues from the cassette

LSIGEGGFWEGTVKGRTGWFPADCVEEVQMRQYDTRHEAEAASASELFQK*

**4) Shank3-N amino acid sequence**

exon 1-12 additional 13 residues from the cassette

MDGPGASAVVVRVGIPDLQQTKCLRLDPTAPVWAAKQRVLCALNHSLQDALNYGLFQPPSRGRAGKFLDEERLLQDYPPNLDTPLPYLEFRYKRRVYAQNLIDDKQFAKLHTKANLKKFMDYVQLHSTDKVARLLDKGLDPNFHDPDSGECPLSLAAQLDNATDLLKVLRNGGAHLDFRTRDGLTAVHCATRQRNAGALTTLLDLGASPDYKDSRGLTPLYHSALGGGDALCCELLLHDHAQLGTTDENGWQEIHQACRFGHVQHLEHLLFYGANMGAQNASGNTALHICALYNQESCARVLLFRGANKDVRNYNSQTAFQVAIIAGNFELAEVIKTHKDSDVVPFRETPSYAKRRRLAGPSGLASPRPLQRSASDINLKGDQPAASPGPTLRSLPHQLLLQRLQEEKDRDRDGELENDISGPSAGRGGHNKISPSGPGGSGPAPGPGPASPAPPAPPPRGPKRKLYSAVPGRKFIAVKAHSPQGEGEIPLHRGEAVKVLSIGEGGFWEGTVKGRTGWFPADCVEEVQMRQYDTRHEAEAASASELFQK*

**2. *Shank3* gKO mice**

**1) PCR product sequencing**

Shank3 exon 12-13 exon 17

TGCTCAGCATTGGGGAGGGCGGTTTCTGGGAGGGAACCGTGAAGGGCCGAACAGGCTGGTTCCCAGCTGACTGTGTGGAAGAAGTGCAGATGCGACAGTATGACACCCGGCATGAAACCAGAGAGGACCGGACGAAGCGTCTCTTCCGCCACTACACTGTGGGTTCCTATGACAGCCTCACTTCACACAGCCCCACCACCCCCAAAGAGGGCTCCCAGCACCACGCTGACCCTGCGGTCCAAGTCCATGACGGCTGAGCTCGAGGAACTTGCTTCCATTCGGAGAAGAAAAGGGGAGAAGTTGGATGAGATCCTGGCAGTTGCCGCAGAG

**2) In-frame codon (stop codon)**

TGCTCAGCATTGGGGAGGGCGGTTTCTGGGAGGGAACCGTGAAGGGCCGAACAGGCTGGTTCCCAGCTGACTGTGTGGAAGAAGTGCAGATGCGACAGTATGACACCCGGCATGAAACCAGAGAGGACCGGACGAAGCGTCTCTTCCGCCACTACACTGTGGGTTCCTATGACAGCCTCACTTCACACAGCCCCACCACCCCCAAAGAGGGCTCCCAGCACCACGCTGACCCTGCGGTCCAAGTCCATGACGGCTGAGCTCGAGGAACTTG...

**3) Protein translation**

exon 12-13 additional 22 residues from exon 17

LSIGEGGFWEGTVKGRTGWFPADCVEEVQMRQYDTRHETREDRTKRLFRHYTVGSYDSLTSHSPTTPKEGSQHHADPAVQVHDG*

**4) Truncated Shank3 amino acid sequence**

exon 1-13 additional 22 residues from exon 17

MDGPGASAVVVRVGIPDLQQTKCLRLDPTAPVWAAKQRVLCALNHSLQDALNYGLFQPPSRGRAGKFLDEERLLQDYPPNLDTPLPYLEFRYKRRVYAQNLIDDKQFAKLHTKANLKKFMDYVQLHSTDKVARLLDKGLDPNFHDPDSGECPLSLAAQLDNATDLLKVLRNGGAHLDFRTRDGLTAVHCATRQRNAGALTTLLDLGASPDYKDSRGLTPLYHSALGGGDALCCELLLHDHAQLGTTDENGWQEIHQACRFGHVQHLEHLLFYGANMGAQNASGNTALHICALYNQESCARVLLFRGANKDVRNYNSQTAFQVAIIAGNFELAEVIKTHKDSDVVPFRETPSYAKRRRLAGPSGLASPRPLQRSASDINLKGDQPAASPGPTLRSLPHQLLLQRLQEEKDRDRDGEQENDISGPSAGRGGHSKISPSGPGGSGPAPGPGPASPAPPAPPPRGPKRKLYSAVPGRKFIAVKAHSPQGEGEIPLHRGEAVKVLSIGEGGFWEGTVKGRTGWFPADCVEEVQMRQYDTRHETREDRTKRLFRHYTVGSYDSLTSHSPTTPKEGSQHHADPAVQVHDG*
